# Transdermal alcohol concentration features predict alcohol-induced blackouts in college students

**DOI:** 10.1111/acer.15290

**Published:** 2024-04-19

**Authors:** Veronica L. Richards, Shannon D. Glenn, Robert J. Turrisi, Kimberly A. Mallett, Sarah Ackerman, Michael A. Russell

**Affiliations:** 1 Edna Bennett Pierce Prevention Research Center, The Pennsylvania State University, University Park, Pennsylvania, USA; 2 Department of Biobehavioral Health, The Pennsylvania State University, University Park, Pennsylvania, USA

**Keywords:** alcohol-induced blackouts, alcohol-related consequences, college students, drinking, transdermal alcohol concentration

## Abstract

**Background:**

Alcohol-induced blackouts (AIBs) are common in college students. Individuals with AIBs also experience acute and chronic alcohol-related consequences. Research suggests that how students drink is an important predictor of AIBs. We used transdermal alcohol concentration (TAC) sensors to measure bio-markers of increasing alcohol intoxication (rise rate, peak, and rise duration) in a sample of college students. We hypothesized that the TAC biomarkers would be positively associated with AIBs.

**Methods:**

Students were eligible to participate if they were aged 18–22 years, in their second or third year of college, reported drinking 4+ drinks on a typical Friday or Saturday, experienced ≥1 AIB in the past semester, owned an iPhone, and were willing to wear a sensor for 3 days each weekend. Students (*N =* 79, 55.7% female, 86.1% White, *M*_age_ = 20.1) wore TAC sensors and completed daily diaries over four consecutive weekends (89.9% completion rate). AIBs were assessed using the Alcohol-Induced Blackout Measure-2. Logistic multilevel models were conducted to test for main effects.

**Results:**

Days with faster TAC rise rates (OR = 2.69, 95% CI: 1.56, 5.90), higher peak TACs (OR = 2.93, 95% CI: 1.64, 7.11), and longer rise TAC durations (OR = 4.16, 95% CI: 2.08, 10.62) were associated with greater odds of experiencing an AIB.

**Conclusions:**

In a sample of “risky” drinking college students, three TAC drinking features identified as being related to rising intoxication independently predicted the risk for daily AIBs. Our findings suggest that considering how an individual drinks (assessed using TAC biomarkers), rather than quantity alone, is important for assessing risk and has implications for efforts to reduce risk. Not only is speed of intoxication important for predicting AIBs, but the height of the peak intoxication and the time spent reaching the peak are important predictors, each with different implications for prevention.

## INTRODUCTION

Alcohol-induced blackouts (AIBs) are a consequence of drinking that act as an accelerant for other consequences (Richards, Glenn, et al., 2023). AIBs are defined as experiencing periods of amnesia for all or part of a drinking episode (Wetherill & Fromme, 2016). Individuals experiencing an AIB remain conscious and continue to engage with their environment. Several studies have shown that AIBs are associated with experiencing additional alcohol-related consequences, ranging in severity (e.g., feeling embarrassed to sexual victimization; Hingson et al., 2016; Merrill et al., 2019; Voloshyna et al., 2018). On nights when an AIB is experienced, college students experience an average of 3.5 additional consequences compared to non-AIB nights (Richards, Glenn, et al., 2023). AIBs have also been associated with longer term consequences such as experiencing symptoms of alcohol use disorder (AUD; Glenn et al., 2023; Studer et al., 2019; Yuen et al., 2021). What makes these statistics even more concerning is the frequency in which student drinkers report experiencing an AIB. In a longitudinal study of more than 1700 college students, approximately 80% reported at least one AIB during college (Glenn et al., 2022). Upon closer examination, these same students experienced an average of eight AIBs during college (Glenn et al., 2023). The frequency of AIBs and associations with additional alcohol-related consequences suggest that reducing AIBs could help prevent alcohol-related harm in college students.

Associations between alcohol consumption and AIBs have been routinely reported in published studies (Evans-Polce et al., 2022; Mallett et al., 2011; Rose & Grant, 2010). It has been shown that quantity of alcohol consumption is not the only contributing factor for AIBs. Several studies have identified the *speed* of intoxication (i.e., rapid rises of blood alcohol concentration) to be an important risk factor for AIBs (Carpenter & Merrill, 2021; White, 2003). Behaviors that impact speed of intoxication (e.g., use of protective behavioral strategies, playing drinking games) have also been associated with AIBs at equivalent levels of alcohol consumption (Carey et al., 2022; Ray et al., 2014; Richards, Turrisi, et al., 2023). Together, these findings indicate that it is not just the amount someone drinks, but *the manner* in which they drink that impacts AIBs.

Nearly all of the literature examining alcohol-related consequences, including AIBs, rely on self-reported drinking data. While self-report provides important information about *how much* someone drinks (and sometimes for how long they drink), it does not provide complete information about the manner in which someone drinks. Concerns have also been raised about using self-report on heavier drinking nights (Northcote & Livingston, 2011). In the context of studying AIBs, self-report is further limited by the notion that individuals do not remember at least part of the drinking episode they are reporting on. Transdermal alcohol concentration (TAC) sensors offer a possible solution by passively measuring biomarkers of alcohol intoxication in near real time that do not rely on self-reports.

The most commonly used TAC sensor has been the SCRAM-Continuous Alcohol Monitor (SCRAM-CAM; Alcohol Monitoring Systems, Littleton, CO). While the SCRAM-CAM has been well validated and used extensively in intervention studies (e.g., Alessi et al., 2017; Barnett et al., 2017; Dougherty et al., 2014, 2015; Mathias et al., 2018), it is limited due to its appearance/size, discomfort, and the stigma associated with wearing an ankle monitor (Courtney et al., 2022; Villalba et al., 2020). Studies using a newer generation, wrist-worn TAC sensor, the BACtrack Skyn (BACtrack Inc., San Francisco, CA) have emerged. These studies indicate the potential to incorporate a valid and more user-friendly TAC sensor into “real-world” alcohol studies (e.g., Ash et al., 2022; Courtney et al., 2022; Richards et al., 2022; Rosenberg et al., 2023; Wang et al., 2021). Evidence supports its validity compared to both the SCRAM-CAM and self-reports (Courtney et al., 2022; Richards et al., 2022). The Skyn uses fuel cell technology to measure TAC every 20 s, resulting in the continuous measure of alcohol intoxication (Wang et al., 2018).

Previous studies have examined all parts of the TAC curve as predictors of outcomes, including those reflective of declining alcohol intoxication (Russell et al., 2022; Simons et al., 2015). For example, fall rate describes the speed of alcohol elimination during declining portions of the curve. Duration (time spent alcohol positive) and area under the curve (AUC; cumulative burden of alcohol exposure; the sum of TAC level by time across the day) combine increasing and decreasing segments of the curve. In this study, we focus on rising portions of the TAC curve, including rise rate (the speed of alcohol intoxication), rise duration (the length of time spent under rising intoxication), and peak TAC (maximum TAC observed). Focus on features associated with rising portions of the TAC curve will translate more effectively to preventive actions because people can change how much and how quickly they drink, but they cannot change how quickly it is eliminated once consumed. Table 1 shows these features, describing their interpretation, operationalization, and calculation.

### This study

This study was an analysis of data from an intensive longitudinal study incorporating the Skyn TAC sensor and daily diaries designed to examine TAC features and AIBs in a “risky” drinking sample of college students across 12 days (Thursday, Friday, and Saturday) over four weekends. We include college students in their second and third years because this is a developmentally important time for alcohol risk (e.g., students move off campus, experience less monitoring/regulation, and approach their 21st birthdays). This study design allows for the examination of TAC features that vary daily, to answer the question: *Does the way in which students drink predict AIBs*? Our hypotheses focused on the day-level since we were interested in how daily differences (rather than average differences between people) in drinking manner predicted AIBs. Previous work demonstrates that days with faster rise rates and higher peaks were significantly associated with reporting more alcohol-related consequences (Russell et al., 2022). No study to the best of our knowledge has examined the relationship between rise duration and any alcohol-related consequences, but previous work showed a positive association between overall duration and alcohol-related consequences (Russell et al., 2022). We aimed to extend this work by examining rising TAC features as a predictor of AIBs. We hypothesized that TAC features associated with the rising portion of the curve (rise rate, peak, and rise duration) would be positively associated with AIBs because they index times in which alcohol intoxication was increasing.

## MATERIALS AND METHODS

### Procedures and participants

The study consisted of 79 participants aged 18–22 years who regularly engaged in “risky” drinking. To recruit the sample, we randomly selected 6000 students (50% sophomores and 50% juniors based on credits) from the university registrar’s database of students at a large, public university in the northeastern United States. Participants received an email invitation describing the study and inviting their participation. All recruitment materials (invitation email and up to six daily reminder emails) included a personalized URL to access a screening survey. Students were eligible to participate if they were aged 18–22 years, in their second or third years of college, reported drinking four or more drinks on a typical Friday or Saturday in the past semester, experienced at least one AIB in the past semester, owned an iPhone, and were willing to wear a sensor for 3 days each weekend (Thursday night through Sunday morning over four weekends). Eligible students were immediately redirected to a baseline survey which took approximately 20 min to complete. At the end of the baseline survey, students scheduled a 10-min appointment to come into the laboratory to pick up the sensor and receive training on how to use it. Training took place the Monday–Thursday prior to the daily portion of the study beginning. Data collection was timed to avoid a large philanthropic event that takes place on campus each February, spring break, and final examination periods in order to reflect typical drinking patterns. All procedures were approved by the University’s Institutional Review Board.

At screening, 15.5% (*n =* 927) of invited students consented to participate in the study, of which 73.1% (*n =* 678) completed the screening. A total of 28.3% (*n =* 192) of screened participants were eligible to continue, of which 100% completed the baseline survey. Due to device availability, 80 students were able to participate in the field period. Prior to the field period, one student dropped from the study, resulting in a final sample of 79 students.

Students were instructed to wear the sensor continuously starting Thursday evening at 5 p.m. through Sunday morning when they woke up for four weekends, with the exception of removing the device to shower. We used the BACtrack Skyn model T15. The sensors can hold a charge for up to 10 days, so students were instructed to charge them once a week prior to Thursday. They received an email and text message at 4 p.m. each Thursday reminding them to turn on the device and wear it by 5 p.m. Daily surveys were sent to all students each weekend morning (Friday–Sunday) at 10 a.m. for four weekends about the day/night before. Surveys were available until 6 p.m. each day. To enhance compliance and retention, students received an email and text reminder 3 days prior to the start of each social weekend (i.e., students received a reminder on Monday to begin on Thursday). An email reminder to complete the survey at 1 p.m. and a text message reminder at 4 p.m. each weekend day was also sent to students. There was an average completion rate of 89.9% across the 12 surveys (range: 1–12).

Participants were compensated $15 for completing the baseline survey and an additional $5 for each survey completed, for up to $75 total. Once participants completed nine surveys, they were entered for a chance to win one of eight $100 gift cards. Completing nine surveys resulted in nine chances per student, with this increasing by two for each additional completed survey (up to 15 chances for completing all 12 surveys).

In the present sample of 79 students, the mean age at baseline was 20.1 years (SD = 0.9) and the majority identified as female (55.7%) and White (86.1%). Participants also identified as Hispanic/Latino (11.4%), Asian (5.1%), Black (1.3%), or multiracial (3.8%). The sample was split evenly between sophomores (49.4%) and juniors (50.6%). Table 2 shows participant demographics by AIB occurrence.

### Measures

#### Alcohol-induced blackouts

The Alcohol-Induced Blackout Measure-2 (ABOM-2; Boness et al., 2022) was used to assess whether students experienced a fragmentary or en bloc AIB each night they reported drinking. Students were asked, “As a result of drinking yesterday, did you/were you ______” Response options were dichotomous with “No” (0) and “Yes” (1). Fragmentary AIBs were assessed with four items (e.g., have fuzzy memories of events, unable to remember a few minutes). En bloc AIBs were assessed with four items (e.g., unable to remember what happened the night before, wake up with no idea where you had been). The eight items from the two types of AIBs were examined together due to low frequency of en bloc AIBs (*n =* 18 en bloc AIBs reported). Responses from the eight items were summed and recoded to a dichotomous scale with “No AIB reported” (0) and “Yes, AIB reported” (1).

#### Segmenting TAC data into “social days”

Because drinking often extends past midnight and is not encapsulated neatly in midnight-to-midnight calendar days, we used “social days” as the primary temporal unit of analysis. As in previous TAC sensor studies of alcohol use, TAC data were segmented into “social days” using 10:00 a.m. as the boundary for the end of the social day because it matched the time of the morning survey (Courtney et al., 2022; Richards et al., 2022; Russell et al., 2022). If participants had any valid TAC data on a social day, it was considered a drinking day. Day-level features were calculated separately for each social day if a TAC drinking episode spanned multiple social days.

#### Day-level TAC data

##### Initial processing

Drinking episodes were identified and coded using a combination of previously published research guidelines (Courtney et al., 2022; Didier et al., 2023; Richards et al., 2022). We applied algorithms to filter out observations in which the sensor was turned on but not worn. These algorithms are based on the sensor’s temperature and movement sensors (similar to algorithms used by Didier et al., 2023). The sensor assesses TAC every 20 s, resulting in 443,999 observations. First, if the temperature sensor indicated a temperature greater than 28°C, it was considered worn (88% of observations). Second, if the temperature was less than or equal to 28°C but more than 5°C above the participants minimum temperature, it was considered worn (an additional 7.4% of observations). Third, if the temperature was more than 3°C above the participant’s minimum temperature and the motion sensor registered above 0.01 Gs, it was considered worn (an additional 0.4% of observations).

##### Identifying drinking episodes

Once nonworn observations were filtered out of the dataset, drinking episodes were identified. Smoothing is fitting a longitudinal function to the data. It is commonly performed with TAC sensor data to minimize the influence of random measurement noise in the time series and facilitate feature extraction (Courtney et al., 2022; Richards et al., 2022; Russell et al., 2022). As a first step, the TAC time series for each person was smoothed using a 30-min centered moving average (e.g., Courtney et al., 2022; Richards et al., 2022). Following recommendations from the manufacturer, observations that were less than 0 after smoothing were recorded to 0. Episodes were demarcated according to the following. The start of an episode was indicated if (a) there were two consecutive zero readings followed by at least one positive (TAC ≥ 5) reading, and (b) there was a positive at the start of the data stream and at least one positive on the next two measurement occasions. The end of an episode was indicated if (a) an alcohol positive is followed by two consecutive nonpositive readings, (b) the last alcohol-positive observation is the last reading for the person, or (c) an alcohol-positive observation is followed by a negative that is the person’s last observation. Episodes were divided in two if consecutive observations were more than 30 min apart. False positive episodes were filtered out using guidelines adapted from previously published studies (Courtney et al., 2022; Didier et al., 2023; Richards et al., 2022). Our specific criteria focused on identifying TAC curves with features that were biologically implausible according to previous literature and manufacturer information. Specifically, we removed episodes that (1) were less than 45 min in duration, (2) were less than or equal to 60 min in duration and with a peak greater than or equal to 400 *μ*g/L air, and/or (3) had rise and fall rates that do not fall between ±20 and 300. This removed 38% of originally identified episodes, but these episodes contained only 6.8% of the total sensor data, leaving 718 drinking episodes with valid TAC data.

##### Calculating day-level TAC features

Three TAC features were extracted from each social day with TAC-positive episode data. If a day contained multiple TAC episodes, features were calculated using all data for the day. TAC features included peak, rise rate, rise duration, and rise AUC. A description of TAC features’ interpretations and calculations are available in Table 1. TAC features were set to zero if the sensor was worn for 80% or more of the hours of the social day but there were no episodes present (*n* = 232 days), as this suggested that no drinking had occurred. TAC features were left missing if no episodes were present but the sensor was worn for less than 80% of the hours of the social day (*n* = 152 days). Of the 152 missing days, daily diaries were completed for 28 days, of which only 12 days were drinking days (*M* drinks reported = 1.74, SD = 2.65).

### Statistical analysis

All data analyses were conducted in R. Means, day-, week-, and person-level SDs were generated from empty multilevel linear models. To test our hypothesis, multilevel logistic models conducted to examine the associations between individual TAC features (rise rate, peak, and rise duration) and AIBs. Each model had three levels of variation (day, week, and person). We used a 3-level centering strategy to partition the variance of each TAC feature into day, week, and person levels. Raw TAC feature values were centered on person-day-means (creating a day-level TAC feature variable), person-week-means were centered on person-means (creating a week-level TAC feature variable), and person-means were centered on the grand mean (creating a person-level TAC feature variable). All variables were *z*-scored to allow for meaningful comparison of odds ratio (OR) effect sizes. Each individual TAC feature model included random intercepts and slopes. A post hoc analysis was conducted to examine one model containing all three TAC features. To account for the strong correlations between rise rate and peak TAC (*r*s between 0.78 and 0.83 at day, week, and person levels; Table S1), we conducted three additional models including a combination of two features per model (i.e., rise rate and rise duration; peak TAC and rise duration; and rise rate and peak TAC). Models were estimated in a Bayesian framework (with noninformative priors and 20,000 total iterations [50% warmup]) using the brms package in R (Bürkner, 2017). Model estimates are the means of the posterior parameter distributions; significance is determined using 95% credible intervals (CIs). Associations are presented as ORs; CIs that do not contain 1.0 were considered significant.

## RESULTS

### Descriptive statistics

Table 3 shows descriptive statistics for study variables among students with valid TAC data (*n* = 76 participants, 410 days with reports on whether or not an AIB was experienced). A total of 486 TAC-positive days were recorded and 147 AIBs were reported. More than two thirds (69.3%) of students experienced at least one AIB. Of those who experienced at least one AIB, an average of 2.2 AIBs (SD = 1.5) were experienced over the 12-day study period.

### Associations between transdermal alcohol concentration features and blackouts

Results of the full multilevel logistic models are presented in Table 4. All TAC drinking features examined predicted AIBs. Days with faster TAC rise rates (Table 4, model 1: OR = 2.69, 95% CI: 1.56, 5.90), higher peak TACs (Table 4, model 2: OR = 2.93, 95% CI: 1.64, 7.11), and longer rise TAC durations (Table 4, model 3 OR = 4.16, 95% CI: 2.08, 10.62) were associated with greater odds of experiencing an AIB.

Results of the models containing combined TAC features are presented in Table 5. When all three features were included, daily rise rate duration remained significantly associated with AIBs, while daily rise rate and peak TAC were not associated with AIBs (Table 5, model 1). In the model containing rise rate and rise duration, both features were associated with AIBs at the day level (Table 5, model 2). In the model containing peak TAC and rise duration, both features were associated with AIBs at the day level (Table 5, model 3). In the model containing rise rate and peak TAC, peak TAC was significantly associated with AIBs but rise rate was not (Table 5, model 4).

## DISCUSSION

More than 30% of all drinking days resulted in an AIB. Full support for our hypothesis was observed. We identified three biomarkers of increasing intoxication (rise rate, peak, and rise duration) that predict likelihood of experiencing an AIB in “risky” college student drinkers. Our results support the notion that the manner in which students drink is important for risk prediction of AIBs.

Days with faster rise rates predicted AIBs. This finding supports the literature which suggests that the speed in which alcohol intoxication increases is important (Carpenter & Merrill, 2021; Goodwin et al., 1969; White, 2003). Carpenter and Merrill (2021) assessed drinking every hour during an episode using ecological momentary assessment (EMA) to examine changes in estimated blood alcohol concentration. While using these real-time methods for measuring self-reported drinking may be more accurate than relying on next morning, retrospective reports, EMA still relies on the participant to respond to prompts while actively drinking. TAC sensors are less burdensome to participants since they measure alcohol intoxication passively. Interventions that target speed of drinking would likely reduce the risk of experiencing an AIB. These might include encouraging students to use more protective behavioral strategies (e.g., alternating alcoholic and nonalcoholic drinks) or engaging in less risky drinking behaviors (e.g., avoiding playing drinking games; Carey et al., 2022; Fairlie et al., 2015; Pearson, 2013; Prince et al., 2013).

Days with higher peak TACs were associated with increased likelihood of AIBs. Reducing the speed of intoxication (rise rate) would also work to decrease peak TAC. Higher peak TACs correspond with higher BACs which are indicative of acute alcohol intoxication (Dougherty et al., 2012; Vonghia et al., 2008). Peak BAC has often been a target for intervention in college students using personalized normative feedback (PNF; e.g., Carey et al., 2007; Collins et al., 2002; Miller et al., 2016). Peak TAC could be emphasized in PNF to facilitate greater precision in treatment (Barnett, 2015).

Days with longer rise durations were associated with increased likelihood of AIBs. Rise duration corresponds to the amount of time spent increasing alcohol intoxication. In conjunction with rise rate and peak, time spent becoming intoxicated can be a target for intervention. Previous work in young adults showed that high intensity drinking days (8+/10+ drinks for females/males) were characterized by longer times spent drinking and more rapid paces of drinking (Patrick et al., 2023). It is possible that targeting reductions in any one of the examined TAC features could result in a reduction in others.

Our results extend previous findings that indicate TAC features independently predict drinking and related consequences (Russell et al., 2022; Simons et al., 2015). We identified three TAC features that are strongly predictive of AIBs (ORs between 2.69 and 4.16). Estimates from models combining these features indicate that rise rate duration contributes the largest amount of unique variance to our prediction. It is unclear if this translates to the “most important” clinical TAC feature, as intervention trials using TAC have not yet been conducted and it is therefore unclear which features are most amenable to change. Future studies are needed to identify the degree to which each TAC feature is amenable to change in intervention efforts.

An alternative approach could have been to examine *all* TAC features available, such as fall rate and AUC. We declined to examine fall rate in relation to AIBs because it is difficult to behaviorally alter alcohol elimination, and may be limited in implications for prevention and intervention efforts. TAC-AUC has been identified as a predictor for alcohol-related consequences (Russell et al., 2022; Simons et al., 2015). A large TAC-AUC may be indicative of several different patterns of drinking. It may reflect a drinking episode with a high peak and short duration or a drinking episode with a low peak but long duration. Thus, it may be difficult to know which part of the drinking curve to directly target for optimal harm reduction. We instead focused on features with clearer implications for prevention and intervention.

### Limitations

The following limitations should be considered. First, our sample consisted of primarily White, female college students who reported recent heavy drinking and AIBs. Results may not generalize to more diverse populations or those with different drinking patterns. Second, the study was conducted over a 1-month period which included St. Patrick’s Day and Easter weekend. St. Patrick’s Day may be associated with especially high risk drinking (Mallett et al., 2013) and Easter may be associated with especially low risk drinking because students tend to travel home for this holiday. The study period is perhaps more representative of a students’ “true” drinking patterns because of this variability. Third, it is possible that the sensors missed lower intensity drinking days (Barnett et al., 2014). There is currently no “gold standard” algorithm for detecting drinking days for the Skyn sensor. Our guidelines for detecting drinking days were informed by previous studies (Courtney et al., 2022; Didier et al., 2023; Richards et al., 2022). Fourth, our study had a analytic sample of 76 students which was chosen, in part, based on costs for the sensors and providing incentives to participants for four weekends of data collection. The sample size did limit the examination of potential moderating effects (e.g., baseline AUD, contextual factors) which may have important implications for risk reduction and prevention (Stevely et al., 2020). Fifth, we were unable to examine the relationship between TAC features and type of AIB because we observed only 18 en bloc AIBs over the study period. Future research is needed to distinguish between types of AIBs. Sixth, a unique aspect of AIBs is that students may not realize they had one the next morning. It is possible that students completed the morning surveys prior to speaking with friends who were present during the reported drinking episode, thus they may misreport the absence of an AIB.

## CONCLUSION

We identified three TAC drinking features related to rising intoxication that independently predicted the risk for daily AIBs among a sample of “risky” drinking college students. Our findings suggest that considering manner in which someone drinks as assessed using TAC biomarkers, rather than quantity alone, is important for risk reduction. Not only is speed of intoxication important for predicting AIBs, but peak intoxication and how long they spend reaching their peak are also important predictors with prevention implications.

## Supplementary Material

Supplemental Table 1

## Figures and Tables

**TABLE 1:** Day-level transdermal alcohol concentration (TAC) features defined.

Feature	Interpretation	Operationalization	Calculation
*Rise rate*	The speed of alcohol consumption/exposure	The day’s average rate of TAC *increase* per hour	$\text{mean}\left(\frac{\Delta {\text{TAC}}_{\text{idt}}}{\Delta {\text{Hours}}_{\text{idt}}}\right)|\Delta {\text{TAC}}_{\text{idt}}>0$
*Peak*	The level of intoxication “achieved”	Maximum TAC observed for the day’s drinking event(s)	max(TAC_idt_)
*Rise duration*	The duration of time spent under rising alcohol intoxication	The total number of hours in which the curve was rising that day	sum(ΔHours_idt_) | ΔTAC_idt_ > 0

**TABLE 2:** Participant characteristics.

	Experienced an AIB (*N* = 59)	Did not experience an AIB (*N* = 20)
	Frequency (%)	Frequency (%)
Sex		
Female	34 (77.3%)	10 (22.7%)
Male	25 (71.4%)	10 (28.6%)
Age		
Mean (SD)	20.2 (0.9)	19.8 (1.0)
Race		
Asian	2 (50.0%)	2 (50.0%)
Black	0 (0.0%)	1 (100.0%)
Multiracial	1 (33.3%)	2 (66.7%)
White	54 (79.4%)	14 (20.6%)
Other	2 (66.7%)	1 (33.3%)
Ethnicity		
Hispanic/Latino	4 (44.4%)	5 (55.6%)
Not Hispanic/Latino	55 (78.6%)	15 (21.4%)
Year in school		
Sophomore	28 (71.8%)	11 (28.2%)
Junior	31 (77.5%)	9 (22.5%)

Abbreviation: AIB, alcohol-induced blackout.

**TABLE 3:** Descriptive statistics for study variables.

	*N* persons	*N* weeks	*N* days	Mean	Person-level SD	Week-level SD	Day-level SD
AIBs^a^	76	224	410	−1.15	0.94	0.39	–
TAC rise rate	78	247	717	53.11	14.09	5.10	53.11
Peak TAC	78	247	718	120.87	66.55	23.48	148.04
Rise TAC duration	78	247	717	2.36	0.54	0.28	2.56

Abbreviations: AIB, alcohol-induced blackout; SD, standard deviation; TAC, transdermal alcohol concentration.

a The AIB empty model was logistic, thus there was no residual (i.e., no day-level SD).

**TABLE 4:** Results from multilevel logistic models predicting alcohol-induced blackouts from transdermal alcohol concentration (TAC) features.

	Model 1: TAC rise rate		Model 2: Peak TAC		Model 3: Rise TAC duration	
	OR	95% CI	OR	95% CI	OR	95% CI
Fixed effects						
Intercept	**0.16**	0.07, 0.27	**0.13**	0.05, 0.25	**0.08**	0.02, 0.18
Daily TAC	**2.69**	1.56, 5.90	**2.93**	1.64, 7.11	**4.16**	2.08, 10.62
Weekly mean TAC	1.32	0.97, 1.90	**1.52**	1.07, 2.23	1.14	0.75, 1.75
Person-mean TAC	**2.24**	1.47, 3.84	**1.86**	1.24, 3.16	**1.91**	1.18, 3.34
Week-level random effects						
Intercept SD	0.82	0.03, 2.17	1.01	0.05, 2.51	1.64	0.51, 2.96
Daily TAC slope SD	1.05	0.08, 2.47	1.17	0.15, 2.66	0.65	0.03, 1.92
Correlation (intercept and daily TAC slope)	0.03	−0.89, 0.93	−0.05	−0.90, 0.92	−0.36	−0.98, 0.84
Person-level random effects						
Intercept SD	0.60	0.04, 1.32	0.77	0.07, 1.63	0.55	0.02, 1.46
Daily TAC slope SD	0.72	0.07, 1.59	0.78	0.06, 1.81	1.13	0.25, 2.11
Correlation (intercept and daily TAC slope)	0.41	−0.79, 0.98	0.31	−0.76, 0.97	−0.07	−0.94, 0.93

*Note*: Significant fixed effects (95% CIs that do not contain 1) are in bold; all independent variables were *z*-scored to allow meaningful comparison of OR effect sizes.

Abbreviations: CI, credible interval; OR, odds ratio; SD, standard deviation; TAC, transdermal alcohol concentration.

**TABLE 5:** Results from multilevel logistic models predicting alcohol-induced blackouts from combined transdermal alcohol concentration (TAC) features.

	Model 1: Rise rate, peak, and rise duration		Model 2: Rise rate and rise duration		Model 3: Peak and rise duration		Model 4: Rise rate and peak	
	OR	95% CI	OR	95% CI	OR	95% CI	OR	95% CI
Fixed effects								
Intercept	**0.09**	0.04, 0.18	**0.10**	0.05, 0.19	**0.11**	0.05, 0.21	**0.18**	0.10, 0.27
Daily TAC rise rate	1.35	0.80, 2.46	**1.58**	1.12, 2.23	–	–	1.19	0.74, 1.99
Weekly mean TAC rise rate	0.97	0.50, 1.81	**1.47**	1.03, 2.15	–	–	0.96	0.58, 1.53
Person-mean TAC rise rate	**2.62**	1.25, 6.48	**2.22**	1.32, 4.03	–	–	**2.03**	1.12, 3.96
Daily Peak TAC	1.23	0.72, 2.16	–	–	**1.53**	1.10, 2.18	**1.87**	1.21, 3.96
Weekly mean Peak TAC	2.02	0.94, 4.75	–	–	**1.91**	1.23, 3.17	1.55	0.95, 2.74
Person-mean Peak TAC	0.77	0.30, 1.73	–	–	1.56	0.82, 3.03	1.00	0.56, 1.70
Daily TAC rise duration	**2.65**	1.61, 4.81	**2.68**	1.77, 4.36	**2.30**	1.45, 3.88	–	–
Weekly mean TAC rise duration	0.72	0.40, 1.24	0.99	0.67, 1.49	0.70	0.42, 1.14	–	–
Person-mean TAC rise duration	1.26	0.61, 2.66	1.11	0.65, 1.89	1.18	0.62, 2.32	–	–
Week-level random effects								
Intercept SD	1.15	0.10, 2.27	0.98	0.06, 2.02	0.97	0.07, 1.98	0.66	0.03, 1.54
Person-level random effects								
Intercept SD	0.79	0.08, 1.57	0.69	0.05, 1.41	0.81	0.09, 1.48	0.71	0.08, 1.32

*Note*: Significant fixed effects (95% CIs that do not contain 1) are in bold; all independent variables were *z*-scored to allow meaningful comparison of OR effect sizes.

Abbreviations: CI, credible interval; OR, odds ratio; SD, standard deviation; TAC, transdermal alcohol concentration.

## Data Availability

The data that support the findings of this study are available from the corresponding author upon reasonable request.
