# Supplementary material for: Transdermal alcohol concentration features predict alcohol-induced blackouts in college students
Source: Alcohol Clin Exp Res (Hoboken). Author manuscript; Available in PMC 2024 May 23. (PMC11114374; doi:10.1111/acer.15290)
Supplement: Supplemental Table 1 [file NIHMS1990311-supplement-Supplemental_Table_1.docx]

| Supplemental Table 1. Correlations between transdermal alcohol concentration features | | | | | |
| --- | --- | --- | --- | --- | --- |
| **Day-Level Correlations (correspondence of days within the same week)** | | | | | |
|  | Peak | | Rise Rate | | Rise Duration |
| Peak | 1 | | 0.83 | | 0.75 |
| Rise Rate | 0.83 | | 1 | | 0.61 |
| Rise Duration | 0.75 | | 0.61 | | 1 |
|  |  |  | |  | |
| **Week-Level Correlations** **(correspondence of weekly averages within the same person)** | | | | | |
|  | Peak | | Rise Rate | | Rise Duration |
| Peak | 1 | | 0.79 | | 0.67 |
| Rise Rate | 0.79 | | 1 | | 0.44 |
| Rise Duration | 0.67 | | 0.44 | | 1 |
|  |  | |  | |  |
| **Person-Level Correlations (correspondence of person averages)** | | | | | |
|  | Peak | | Rise Rate | | Rise Duration |
| Peak | 1 | | 0.78 | | 0.8 |
| Rise Rate | 0.78 | | 1 | | 0.57 |
| Rise Duration | 0.8 | | 0.57 | | 1 |
